# Effect of Vulcanization Process Parameters on the Tensile Strength of Carcass of Textile-Rubber Reinforced Conveyor Belts

**DOI:** 10.3390/ma14247552

**Published:** 2021-12-09

**Authors:** Tsegaye Sh. Lemmi, Marcin Barburski, Adam Kabzinski, Krzysztof Frukacz

**Affiliations:** 1 Faculty of Material Technologies and Textile Design, Institute of Architecture of Textiles, Lodz University of Technology, 90-924 Lodz, Poland; Marcin.barburski@p.lodz.pl; 2 Sempertrans Bełchatów Sp. z o.o., 97-427 Rogowiec, Poland; Adam.Kabzinski@semperitgroup.com (A.K.); Krzysztof.Frukacz@semperitgroup.com (K.F.)

**Keywords:** woven fabric, tensile strength, aging, carcass, dipping, rubber, vulcanization, conveyor belt

## Abstract

Textile-reinforced conveyor belts are most widely used in various industries, including in the mining, construction, and manufacturing industries, to transport materials from one place to another. The conveyor belt’s tensile strength, which primarily relies on the property of the carcass, determines the area of application of the belt. The main aim of the current work was to investigate the influence of vulcanization temperature and duration of the vulcanization process on the tensile properties of the carcass part of the conveyor belt. An extensive experiment was carried out on the tensile properties of woven fabrics that were intended to reinforce conveyor belts by aging the fabrics at the temperature of 140 °C, 160 °C, and 220 °C for six and thirty-five minutes of aging durations. Afterward, the textile-reinforced conveyor belts were produced at vulcanization temperatures of 140 °C, 160 °C, and 220 °C for six and thirty-five minutes of vulcanizing durations. The influence of the vulcanization process parameters on the tensile property of fabrics utilized for the reinforcement of the conveyor belt was analyzed. In addition, the effect of the dipping process of woven fabric in resorcinol–formaldehyde–latex on the tensile property of polyester/polyamide 66 woven fabric (EP fabric) was investigated. The investigation results revealed that the tensile strength of the carcass of the conveyor belt was significantly affected by vulcanization temperature. The conveyor belt vulcanized at 160 °C for 35 min has shown the optimum tensile strength, which is 2.22% and 89.06% higher than the samples vulcanized at 140 °C and 220 °C for 35 min, respectively. Furthermore, the tensile strength and percentage elongation at break of conveyor belts vulcanized at 220 °C were almost destroyed regardless of the vulcanization duration.

## 1. Introduction

The demand for bulk materials transportation with higher efficiency and affordable transportation cost expedites the revolution of conveyor belt technology. Since the first conveyor belt was used in the early 19th century [1], a lot has been changed; the invention of vulcanized rubber by Charles Goodyear in 1839 [2] and the introduction of thermoplastic fibers to the market in the mid-20th century has significantly boosted the development of a textile–rubber reinforced conveyor belt production sector.

Conveyor belts are used for the continuous transport of lightweight to heavy materials over short to long distances. The belt’s construction and properties are determined based on its area of application. The heavy-duty conveyor belts are employed to convey bulk materials in mining, construction, agriculture, power, and other industries [3,4,5,6,7]. The construction of the conveyor belt comprises three main components: the carcass, skim, and covers (top and bottom covers). The carcass is a reinforcement part found inside the conveyor belt, and it can be either a textile or steel cord material. The carcass is responsible for providing the required tensile strength of the belt. The skim is a polyvinyl chloride (PVC), rubber, or urethane material used between the plies of the conveyor belt. The covers are rubber materials primarily used in conveyor belt construction to protect the carcass. Additionally, the top and bottom covers of the belt provide necessary wear resistance and adequate friction to the drive pulley, respectively.

The properties of the conveyor belt’s constituent materials and quality of ply adhesions substantially influence the performance, durability, and safety of the conveyor belt system [7,8,9,10]. Furthermore, the processing parameters of the conveyor belt have a significant effect on determining the properties of a conveyor belt. Several researchers have carried out various studies in order to establish optimized conveyor belt construction and processing parameters. Chou et al. [8,11] used a Taguchi method to determine optimum conditions for vulcanizing textile-reinforced conveyor belts with better elongation, adhesive strength, and abrasion resistance. Amr et al. [12] also investigated the effect of the number of plies of the reinforcing materials, loading direction, and speed on the tensile strength of conveyor belts reinforced with a woven fabric using the Taguchi method. The study shows that the tensile strength of the conveyor belt was severely affected by the loading direction and followed by the number of reinforcement plies and loading speed. The increase in the number of carcass plies increases the strength of conveyor belts, which increases the conveyor belt’s thickness and potentially reduces the flexibility of the belt. Ambriško et al. [13] analyzed the effect of carcass, nominal strength, and the number of plies on the tensile strength of textile–rubber reinforced conveyor belts using the Design of Experiment (DOE) method. Rawdha et al. [14] investigated the moisture ingression behavior of the textile-reinforced conveyor belt; the study indicates that the textile carcass is sensitive to moisture ingress, which could affect the durability of the belt. Rudawska et al. [15] studied the effect of temperature and humidity on the mechanical properties of the textile-reinforced conveyor belts by testing the samples in a climatic and thermal shock chamber, and the study reveals that the thermal shock deteriorates the mechanical properties of the conveyor belt. Fedorko et al. [16,17,18] studied in their papers the deterioration of the internal structure of textile reinforced multi-ply conveyor belts due to tensile load and dynamic wear using metro-tomography.

The application of woven fabric as reinforcement material in the conveyor belt is tremendously increasing because of its lightweight, high strength, flexibility, and corrosion resistance properties. These draw the attention of researchers to study how the structure and properties of the woven fabric can influence the properties of a conveyor belt. Barburski et al. [19] analyzed the effect of heat treatment on the internal structure of woven fabrics used for reinforcement of the conveyor belt, and the result indicates that the impact of thermal treatment on the physical properties of the fabric depends on the weave structure of the fabric and duration of thermal treatment. The influence of the fabric’s weave structure on the mechanical properties of the multi-layer woven fabrics used for conveyor belt reinforcement was also investigated by Witczak et al. [20]. The effect of thermal aging parameters on the physical and mechanical properties of the yarns used to produce a carcass of the textile-reinforced conveyor belt was investigated by Lemmi et al. [21]; the study revealed that the percentage elongation of the polyester yarns was adversely affected under high thermal aging temperature with a minimum aging duration, and the yarn’s tenacity has deteriorated at higher aging temperature (220 °C). Kabzinski [22] presented basic information about the conveyor belt design and woven fabric structure used for the purpose of conveyor belt reinforcement.

In order to produce a conveyor belt with the desired physical and mechanical properties, the components of the conveyor belt need to be laminated together. Therefore, the conveyor belt’s components are subjected to a vulcanization process to adhere the constituent materials together. The vulcanization process of the conveyor belt depends on three crucial parameters: vulcanization temperature, time, and pressure [23]. These parameters are primarily adjusted based on the type of rubber, carcass, the thickness of the conveyor belt, and the number of plies.

Even though various studies were carried out on the conveyor belt and constituent parts of the conveyor belt, the effect of the vulcanization process on the mechanical property of the carcass of the conveyor belt has been left behind, despite its cruciality in determining the entire mechanical property of the belt. Therefore, the main objective of this study is to investigate the effect of vulcanization parameters, mainly vulcanization temperature and time, on the tensile property of the textile carcass of the conveyor belt.

## 2. Materials and Methods

### 2.1. Materials

**Figure 1:**
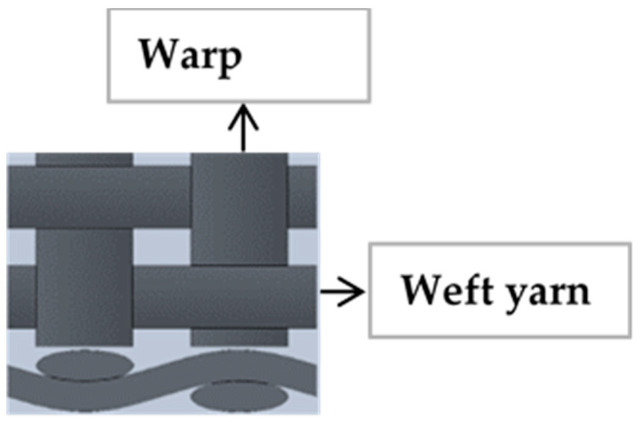
Plain weave pattern.

The fabric woven from high-tenacity polyester and polyamide 66 yarns in the warp and weft direction respectively were supplied from Kordárna Plus a.s. company, Velká nad Veličkou, Czech Republic. This type of fabric is known with an acronym of EP fabric in the conveyor belt manufacturing industries. The fabrics were woven with a plain weave structure. Plain weave is the simplest type of woven fabric structure with an interlacement of warp and weft yarns at the right angle to each other, as shown in Figure 1. Plain weave and its derivates are mainly used to reinforce conveyor belts [22]. Ninety percent of technical fabrics are produced by a plain weave because of its maximum number of binding points and relatively high fabric strength compared to other weave structures [24].

**Figure 2:**
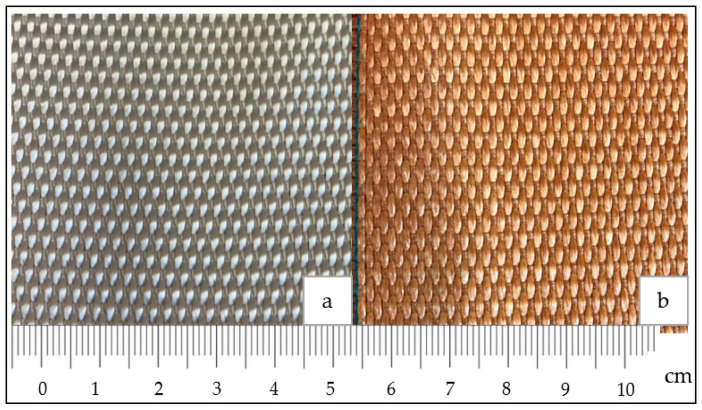
EP 200 woven fabric samples. (**a**) Greige fabric; (**b**) Dipped fabric.

The woven fabrics were supplied by a manufacturer in a greige and dipped form. The greige woven fabrics are fabrics without any finishing treatments, as shown in Figure 2a. The adhesion between the textile reinforcement (carcass) and rubber matrix is necessary to obtain the desired properties of the conveyor belt. However, EP fabrics have no sufficient adhesion property to the rubber material. Therefore, to enhance the adhesiveness of woven fabrics to the rubber, the fabrics were impregnated with a resorcinol–formaldehyde–latex (RFL) solution. The fabric sample dipped in the RFL solution is referred to as dipped woven fabric in this article; the image of the dipped fabric is shown in Figure 2b. The properties of the dipped woven fabric sample used for this study are provided in Table 1.

### 2.2. Methods

Due to the complicated structural composition of the conveyor belt, ascertaining the effect of vulcanization process parameters on the tensile property of the carcass of the conveyor belt is demanding. To determine these effects, the following approaches were used in this study. First, the EP woven fabrics intended to reinforce conveyor belts were subjected to thermal aging in an industrial electric oven under various aging conditions. Subsequently, the multi-ply conveyor belt reinforcements were made using similar woven fabric samples that were thermally aged in the oven. The vulcanization temperature and duration of the process to vulcanize the conveyor belt reinforcements were the same as the parameters used to age the fabrics in the oven. Finally, post thermal aging of the fabric and vulcanization of the conveyor belt, the tensile strength experimental test was carried out on the samples.

**Figure 3:**
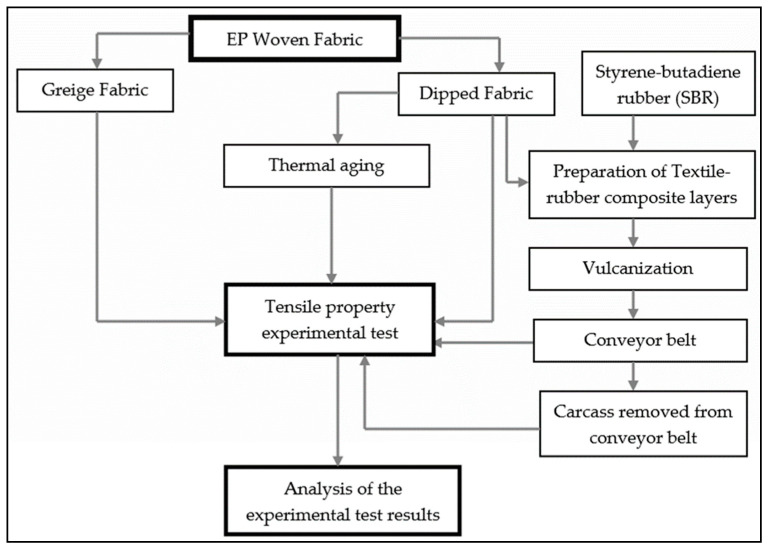
Experimental research flowchart.

Additionally, the woven fabric layers were removed from the conveyor belts, and the tensile strength of these fabrics were also investigated. In addition, the effect of the dipping process of fabric in the resorcinol–formaldehyde–latex (RFL) on the tensile strength and elongation of the woven fabric was studied. The experimental test results at each stage of the process were analyzed to understand the effect of temperature and duration of the vulcanization process on the tensile property of the textile carcass of the conveyor belt. The experimental processes implemented in this work are shown in Figure 3. Moreover, the detailed processes and parameters utilized to fulfill these approaches are also presented in the following sections.

#### 2.2.1. Thermal Aging of Woven Fabric Samples

**Figure 4:**
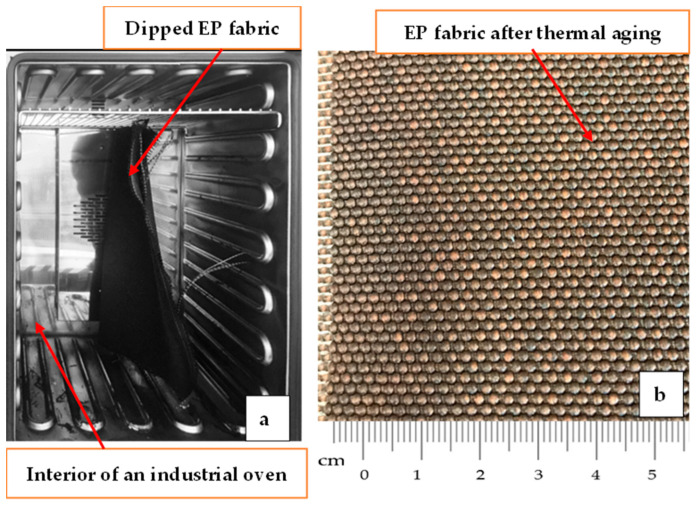
(**a**) An industrial oven for a thermal aging of fabric; (**b**) EP fabric sample aged at 220 °C for 6 min.

The effect of thermal aging on the tensile properties of industrial woven fabrics intended to reinforce conveyor belts was investigated by conducting experimental tests on the woven fabric samples. The fabric samples were thermally aged in an industrial electric oven, as shown in Figure 4 at 140 °C, 160 °C, and 220 °C for six and thirty-five minutes of aging duration. The aging conditions were chosen based on the vulcanization temperatures used in the conveyor belt manufacturing companies, the dipping of the fabrics, and the glass transition temperature of the fiber types in the woven fabric. Subsequent to the thermal aging of the fabric, the samples were relaxed for 24 h at the standard laboratory conditions and subjected to the tensile property test. The details of the fabric samples’ thermal aging conditions are provided in Table 2.

#### 2.2.2. Preparation of a Conveyor Belt Reinforced with a Woven Fabric

Multi-layer conveyor belts reinforced with the woven fabric were prepared under different vulcanization conditions, as shown in Table 3. The conveyor belts were prepared with three layers of EP 200 woven fabric reinforcement; the woven fabric dipped in the RFL solution was used for reinforcement purposes.

**Figure 5:**
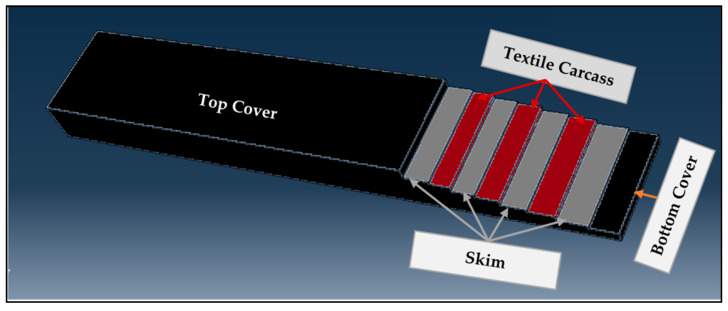
Three-dimensional (3D) model of textile-reinforced multi-ply conveyor belt.

**Figure 6:**
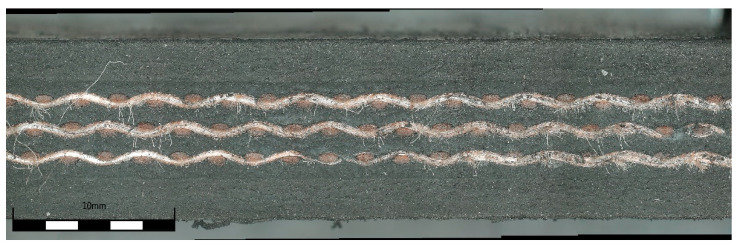
Side view of textile-reinforced conveyor belt made at vulcanization condition of 160 °C for 35 min.

A styrene–butadiene rubber (SBR) was used for the top and bottom covers of the belt. Additionally, SBR synthetic rubber was also utilized as the skim part of the conveyor belt to adhere the plies of the reinforcement. The three-dimensional model of the textile-reinforced conveyor belt is shown in Figure 5. All conveyor belt samples were prepared based on the presented model at different vulcanization conditions. For example, the side view of a textile-reinforced conveyor belt made at vulcanization conditions of 160 °C for 35 min is shown in Figure 6.

#### 2.2.3. Tensile Strength Test of Fabric and Conveyor Belt Samples

**Figure 7:**
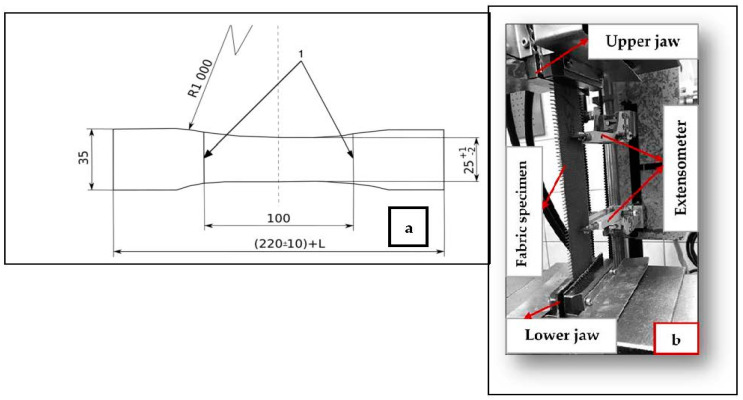
(**a**) Specimen dimensions for conveyor belt tensile testing; (**b**) Tensile strength testing machine.

The tensile properties of greige fabrics, dipped fabrics before and after thermal aging, and conveyor belts were investigated. The tensile strength of the fabric and conveyor belt samples were tested according to ISO 13934-1:2013 [25] and ISO 283:2015 [26], respectively. The samples were tested on a Zwick/Roell tensile testing machine of a 150 kN load cell (Figure 7b) with a mechanical extensometer and a testing speed of 100 mm/min under standard laboratory conditions. The specimens were gripped in the movable upper jaw and stationary lower jaw of the tensile testing machine. For each type of sample, ten specimens were tested, and the test results were analyzed using testXpert II software. A strip method of specimen preparation was used for fabric specimen preparation. The conveyor belt specimens were prepared according to ISO 283:2015 [26], as shown in Figure 7a.

## 3. Results and Discussion

The tensile strength of the EP fabric and textile reinforced conveyor belts under various thermal aging and vulcanization conditions have been investigated and discussed in the following sections.

### 3.1. Tensile Property of Greige and Dipped EP Woven Fabric

In order to analyze the effect of dipping process on the tensile property of the woven fabric, the tensile properties of the woven fabrics at the greige level and after dipping the fabric in RFL solution were investigated. There is no doubt that any type of conveyor belt has to have a proper tensile strength to withstand the maximum load exerted on the belt during the operation throughout its service life. The tensile strength of the belt primarily relies on the properties of a material used to reinforce the belt in a longitudinal direction; when it comes to textile reinforced conveyor belts, the tensile strength of the belt is determined by the tensile property of the fabric in the warp direction.

**Figure 8:**
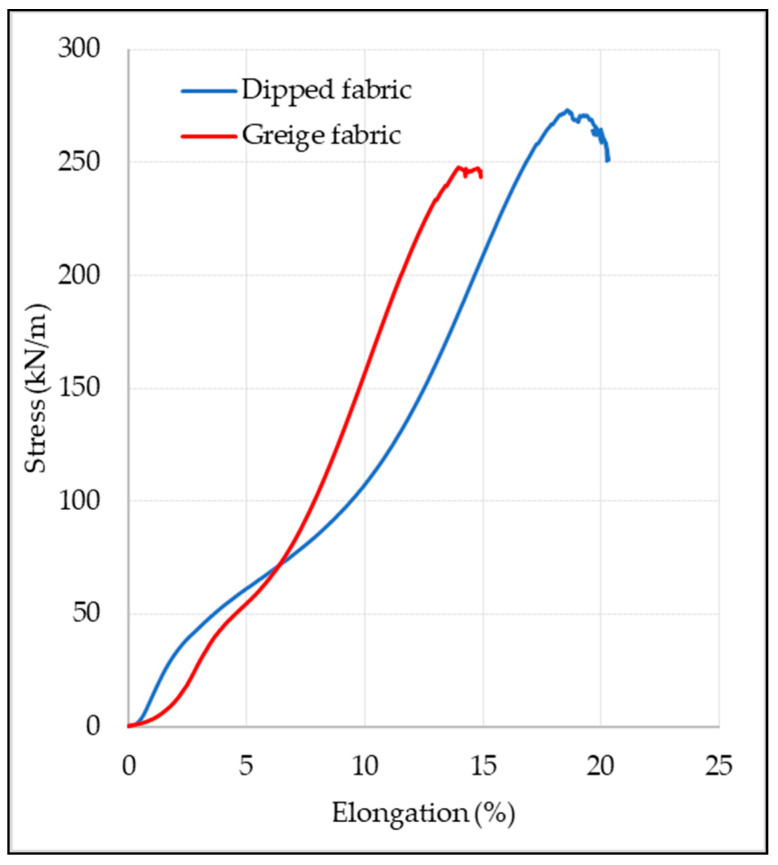
Stress-percentage elongation curve of EP200 greige and dipped fabric samples.

The effect of dipping process on the tensile strength and percentage elongation of the EP woven fabric in a longitudinal direction is shown in Figure 8. The result shows that impregnation of the fabric with the RFL solution has significantly influenced the fabric’s tensile strength and percentage elongation at break. After dipping the fabric in RFL solution, the tensile strength and percentage elongation of the fabric in a warp direction was increased by 11.41% and 30.51%, respectively. In addition, the result reveals that the fabrics were elongated differently under constant stress, as shown in Figure 8.

The stress–strain curve shows that at the beginning of the curve, greige fabric has shown higher elongation. However, as the stress increased, the elongation of dipped fabric surpassed the greige fabric’s elongation. This was because of two fundamental reasons; in the elastic region of the curve, the greige fabric was more elongated due to the high crimp percentage of the greige fabric. Nevertheless, once the warp crimp was removed, the elongation of the fabric was subsequently reduced in comparison to the dipped fabric. The other reason was that the dipped fabric has a stiffer appearance, and its ability to elongate under minor stress was low, but as the stress increases, the elongation of the fabric increases; this signifies that the dipping of EP fabric in resorcinol–formaldehyde–latex (RFL) solution has an influence on the mechanical properties of the fabric. Additionally, it can also be seen that the slopes of these curves are quite far apart from each other, which indicates that the dipping of fabric in RFL has an impact on the elastic modulus of the fabric.

### 3.2. Effect of Thermal Aging on the Tensile Strength of the Woven Fabric

**Figure 9:**
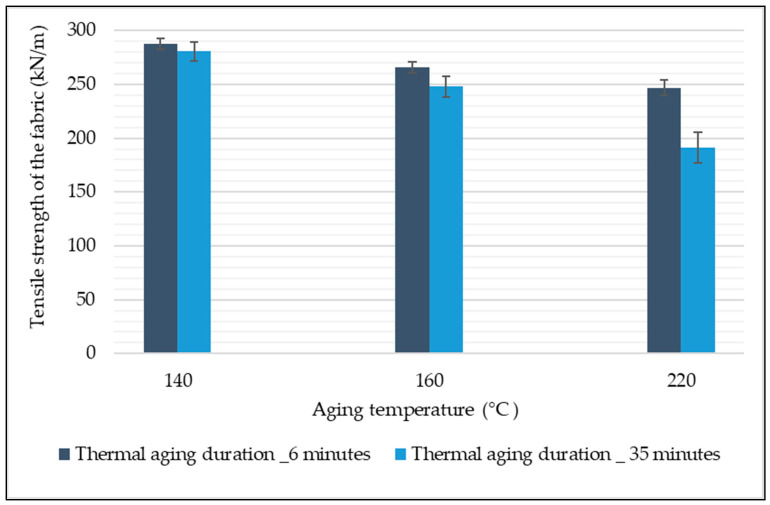
Effect of thermal aging on the tensile strength of RFL-dipped EP woven fabric.

The impact of thermal aging temperature and duration of aging on the tensile properties of EP woven fabrics intended to be used as a carcass of the textile-reinforced conveyor belt were investigated. The tensile strength of thermally aged woven fabrics along with the respective aging temperature and duration of aging are shown in Figure 9. The results revealed that the increases in aging temperature decreased the tensile strength of the fabric regardless of aging duration.

The rise of aging temperature from 140 to 160 °C for the aging duration of 6 min and 35 min decreased the fabric tensile strength by 8.25% and 11.62%, respectively. The increase in aging temperature from 160 to 220 °C for the aging time of 6 min and 35 min decreased the fabric tensile strength by 7.05% and 22.83%. This shows that the rise in thermal aging temperature influenced the tensile strength of the EP fabric.

Additionally, the fabric samples aged at the same thermal aging temperature with different aging durations have decreased the tensile strength of the fabric as the aging duration increases. For example, as the aging duration increased from 6 to 35 min, the tensile strength of fabric aged at 140 °C decreased by 2.42%, while the tensile strength of fabric aged at 160 °C and 220 °C decreased by 11.62% and 22.83%, respectively. From the results, it can be concluded that the mechanical properties of the EP fabric are dependent on the thermal aging temperature and time; in determining the tensile properties of the fabric, the fabric’s fiber composition plays a major role. EP fabric is a composition of yarn made from polyester fiber and polyamide 66; these fibers’ mechanical properties depend on the aging temperature [21,27].

### 3.3. Effect of Thermal Aging Parameters on the Percentage Elongation of Woven Fabric

**Figure 10:**
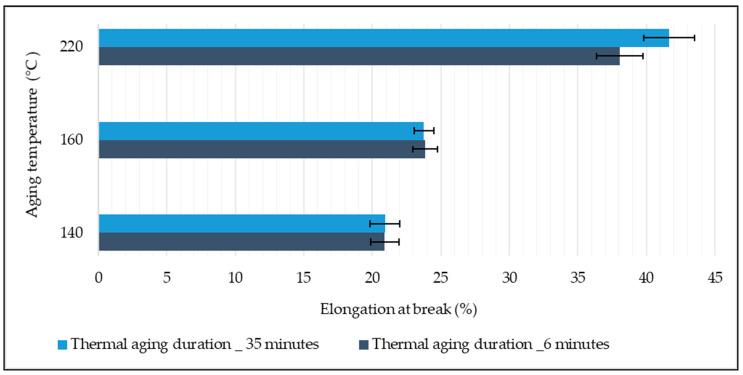
Effect of thermal aging on the elongation property of woven fabric.

The influence of thermal aging on the percentage elongation at break of the EP woven fabrics is shown in Figure 10. The results show that the aging temperature highly impacted the elongation property of the woven fabric for the fabric samples that underwent thermal aging above the glass transition temperature of the polyester fiber. There were no significant changes observed in the percentage elongation at break of fabric samples aged at 140 °C and 160 °C because of the variation of aging duration. However, the fabric samples that underwent thermal aging at 220 °C have shown an 8.59% increase in percentage elongation as the aging time increased from 6 to 35 min. The increment of aging temperature from 140 to 220 °C increased the elongation of the fabric irrespective of aging duration. The highest percentage elongation at break was registered for the samples aged at 220 °C for longer aging time (35 min), which is 49.78% higher than the samples aged at 140 °C.

### 3.4. Effect of Vulcanization Conditions on the Tensile Strength of Carcass of Conveyor Belt

**Figure 11:**
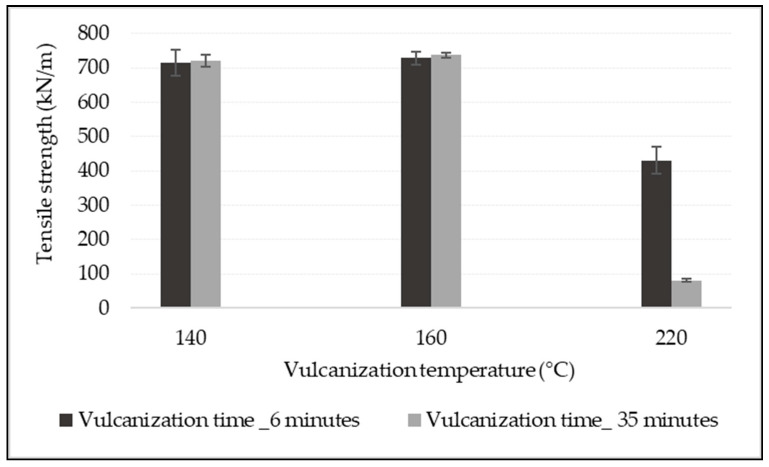
Effect of vulcanization conditions on the tensile strength of conveyor belt.

The carcass of the conveyor belt is the backbone of the belt in determining the tensile property of the belt. The influence of vulcanization temperature and time on the conveyor belt’s tensile strength has been investigated. A multi-layer conveyor belt reinforced with a textile fabric of EP 200N has been vulcanized under three different vulcanization temperatures for six and thirty-five minutes of vulcanization time, while the other vulcanizing parameters were constant. As shown in Figure 11, it is clear that vulcanizing EP fabric-reinforced conveyor belt at high temperatures has extremely affected the tensile property of the belt. The tensile strength investigation results indicate that vulcanizing EP fabric-reinforced conveyor belts at the temperature of 140 °C and 160 °C regardless of vulcanizing time used in this experiment have not shown any considerable tensile strength difference. However, the tensile strength of the conveyor belt vulcanized at 220 °C for 35 min was almost destroyed. Compared to the conveyor belt sample vulcanized at 160 °C, the tensile strength of the samples vulcanized at 220 °C for 35 min has been reduced by 89.06%. In addition to that, the tensile strength of samples vulcanized at 220 °C for 6 min was also reduced by 40.16% compared to conveyor belt vulcanized at 160 °C for 6 min. These results show that the vulcanization of EP fabric-reinforced conveyor belt at high temperature deteriorates the tensile strength of the conveyor belt regardless of vulcanization time; this shows that vulcanizing of the EP-reinforced conveyor belt above the glass transition of the polyester and polyamide 66 fibers can fully deteriorate the tensile property of the belt.

### 3.5. Effect of Vulcanization Conditions on the Percentage Elongation of the Textile Carcass

The elongation properties of a conveyor belt are crucial to determine heavy-duty belts’ performance when subjected to varying stress levels. In conveyor belt design, the low extension of the belt is recommended to increase the belt’s service life, reduce the fluctuation of the power on the drive sharing of rollers, and prevent the driving motor from burning out because of unbalanced drive sharing [28].

**Figure 12:**
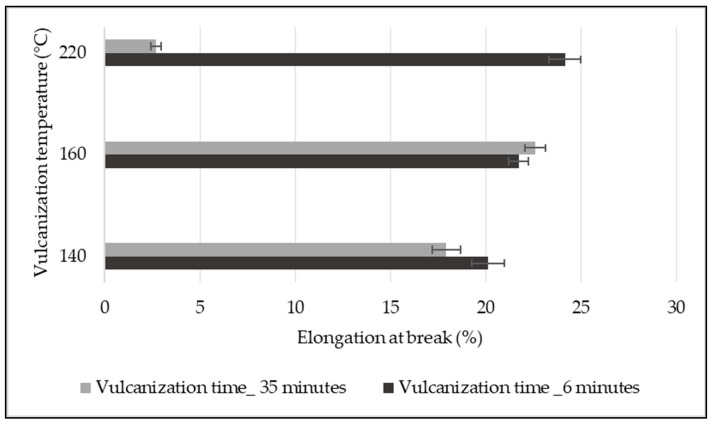
Effect of vulcanization conditions on the percentage elongation of the conveyor belt.

The experimental test results show that the vulcanization temperature and time have an impact on determining the elongation property of a conveyor belt. As shown in Figure 12, the percentage elongation of a conveyor belt vulcanized for six minutes increased with vulcanizing temperature. In addition, the highest percentage elongation of the belt was observed for the sample vulcanized at high temperature (220 °C) for the shortest vulcanizing time (6 min), which was 20.02% higher than the conveyor belt sample vulcanized at 140 °C for 6 min. In contrast, the lowest percentage elongation of the conveyor belt was obtained for the samples vulcanized at 220 °C for thirty-five minutes of vulcanizing time, and this result was 84.95% lower than the samples vulcanized at 140 °C. The carcass of the conveyor belt vulcanized at 220 °C for 35 min was nearly destroyed, and the sample had no ability to withstand the stress imposed on the sample; this was the main reason for the droppage of the percentage elongation and tensile strength of the conveyor belt. Even though lower elongation of the conveyor belt was achieved at the vulcanization condition of 220 °C for 35 min, this cannot be considered as an optimum vulcanization temperature for the conveyor belt design due to its low tensile strength.

### 3.6. Comparison of the Effect of Temperature on Tensile Properties of Woven Fabric and Conveyor Belt

**Figure 13:**
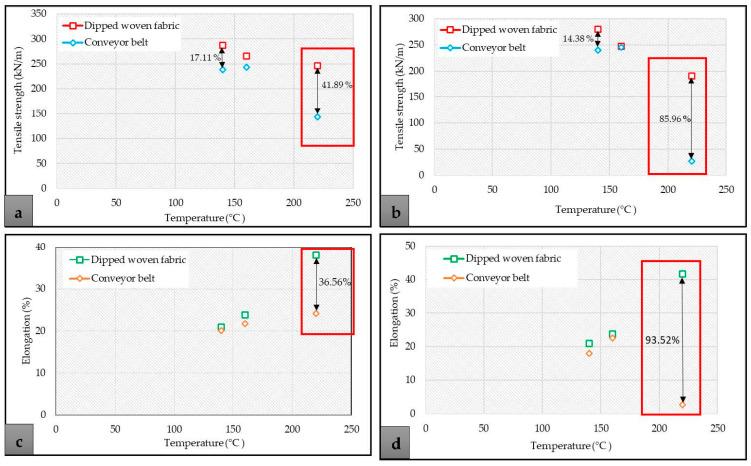
Comparison of tensile strength of thermally aged woven fabric with single ply of the conveyor belt’s carcass. (**a**) Tensile strength of dipped woven fabric and conveyor belt that underwent thermal aging for 6 min; (**b**) Tensile strength of dipped woven fabric and conveyor belt that underwent thermal aging for 35 min; (**c**) Percentage elongation of dipped woven fabric and conveyor belt that underwent thermal aging for 6 min; (**d**) Percentage elongation of dipped woven fabric and conveyor belt that underwent thermal aging for 35 min.

**Figure 14:**
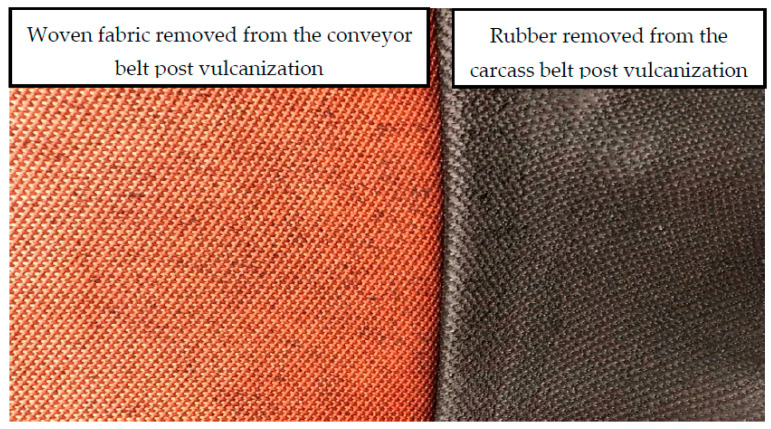
Woven fabric ply and rubber removed from the conveyor belt reinforcement.

The effect of vulcanization temperature and duration of vulcanization process on the tensile property of the conveyor belt’s carcass was compared with the tensile property results of dipped woven fabric aged in the electric oven. The influence of processing parameters on the tensile strength and percentage elongation of the woven fabrics are shown in Figure 13a–d. As mentioned in the previous sections, the conveyor belts with three layers of EP 200 woven fabric were produced. However, to compare the tensile property difference of the fabric thermally aged in the oven with the tensile property of fabric vulcanized with the rubber, the carcass part of the conveyor belt was removed from the reinforcement, as shown in Figure 14.

As shown in Figure 13a–d, even though the fabrics were subjected to thermal aging in different technics under similar conditions, the property of fabrics aged in the oven is less affected in comparison to the vulcanized fabric irrespective of the duration of aging/vulcanization process. This difference arose from the following things: the fabrics aged in the oven were aged under no pressure, but the fabric used as the carcass of the conveyor belt was reinforced under high pressure; this influences the property of the vulcanized fabric. The other reason was that as the temperature of vulcanization increased to 220 °C, the tensile strength of the vulcanized fabric was almost destroyed regardless of the duration of the vulcanization process; this can also be linked with the chemical crosslinking of conveyor belt reinforcement components at high temperature.

Nevertheless, the present study shows that it is not recommendable to vulcanize the EP woven fabric-reinforced conveyor belts at higher temperature (220 °C) even if the duration of the vulcanization process is short unless a special rubber material is used for the reinforcement.

## 4. Conclusions

The present work investigated the effect of vulcanization temperature and duration of the vulcanization process on the carcass of the textile-reinforced conveyor belt under various vulcanizing parameters. Additionally, the influence of dipping of woven fabric in resorcinol–formaldehyde–latex (RFL) and thermal aging parameters on the tensile properties of polyester/polyamide 66 (EP) fabric was analyzed. The following conclusions can be drawn from the study.

- In addition to enhancing the adhesion between the EP fabric and rubber material, the dipping of woven fabric in RFL significantly influenced the fabric’s tensile strength and elastic modulus.
- The tensile strength of the RFL-dipped EP fabric was significantly influenced by the thermal aging parameters. Therefore, the analysis of the study shows that as the aging temperature increases, the tensile strength of the fabric is reduced; moreover, the change in the tensile strength and elongation of the fabric is primarily dependent on the duration of thermal aging.
- In contrast to tensile strength, elongation at break of the fabric is directly proportional to the aging temperature and duration of aging. Nevertheless, for the EP woven fabric samples subjected to thermal aging at 140 °C and 160 °C, there was no considerable change observed on the elongation property of the fabric regardless of the aging time.
- The tensile strength and percentage elongation results of EP fabric-reinforced conveyor belt at the temperature of 140 °C and 160 °C irrespective of vulcanizing time used in this experiment have not shown any considerable changes in the tensile strength and percentage elongation at break. However, the tensile strength and percentage elongation of the conveyor belts vulcanized at 220 °C for 35 min was almost destroyed.

Generally, in textile-reinforced conveyor belt technology, the polyester/polyamide 66(EP) fabrics used as a carcass of the belt either in the single or multi-ply form are highly vulnerable to the vulcanization process parameters. Even though the present work was focused only on the effect of temperature and duration of vulcanization time, there are various parameters that need to be taken under consideration and need further investigation in the future on the dependency of conveyor belt mechanical properties and processing parameters.

## Figures and Tables

**Table 1:** Properties of dipped woven fabric sample.

Fabric Type	Fabric Properties						
	Warp Yarn	Weft Yarn	Warp Count Ends/cm	Weft Count Picks/cm	Mass per Unit Area (g/m^2^)	Crimp of Warp (%)	Weave Type
EP 200 *	Polyester	PA 66	9.10 ± 0.25	4.50 ± 0.15	631 ± 10	2.50	Plain weave

* E-polyester yarn in the warp direction, P-polyamide 66 in the weft direction, 200-nominal strength of the fabric in kNm^−1^.

**Table 2:** Thermal aging conditions of woven fabric samples.

Fabric Type		Aging Conditions	
	Sample	Aging Temperature (°C)	Aging Duration (min)
EP 200-Dipped fabric	01	140	6
	02	140	35
	03	160	6
	04	160	35
	05	220	6
	06	220	35

**Table 3:** Vulcanization conditions of textile-rubber reinforced multi-ply conveyor belt.

			Vulcanization Conditions			
Sample	Carcass Type	Number of Plies	Temperature (°C)	Duration (min)	Pressure of the Press Machine (kN)	Belt Thickness (mm)
01	EP	3	140	6	1020	10
02	EP	3	140	35	1020	10
03	EP	3	160	6	1020	10
04	EP	3	160	35	1020	10
05	EP	3	220	6	1020	10
06	EP	3	220	35	1020	10

## Data Availability

The data presented in this study are available on the request from the corresponding author.
